# Application of Shape Memory Alloys in Retrofitting of Masonry and Heritage Structures Based on Their Vulnerability Revealed in the Bam 2003 Earthquake

**DOI:** 10.3390/ma14164480

**Published:** 2021-08-10

**Authors:** Alireza Tabrizikahou, Marijana Hadzima-Nyarko, Mieczysław Kuczma, Silva Lozančić

**Affiliations:** 1 Institute of Building Engineering, Poznan University of Technology, Piotrowo 5, 60-965 Poznan, Poland; mieczyslaw.kuczma@put.poznan.pl; 2 Faculty of Civil Engineering and Architecture Osijek, Josip Juraj Strossmayer University of Osijek, Vladimira Preloga, 31000 Osijek, Croatia; mhadzima@gfos.hr

**Keywords:** masonry structures, heritage, buildings, earthquake, vulnerability, shape memory alloys, seismic, retrofitting

## Abstract

For decades, one of the most critical considerations of civil engineers has been the construction of structures that can sufficiently resist earthquakes. However, in many parts of the globe, ancient and contemporary buildings were constructed without regard for engineering; thus, there is a rising necessity to adapt existing structures to avoid accidents and preserve historical artefacts. There are various techniques for retrofitting a masonry structure, including foundation isolations, the use of Fibre-Reinforced Plastics (FRPs), shotcrete, etc. One innovative technique is the use of Shape Memory Alloys (SMAs), which improve structures by exhibiting high strength, good re-centring capabilities, self-repair, etc. One recent disastrous earthquake that happened in the city of Bam, Iran, (with a large proportion of masonry buildings) in 2003, with over 45,000 casualties, is analysed to discover the primary causes of the structural failure of buildings and its ancient citadel. It is followed by introducing the basic properties of SMAs and their applications in retrofitting masonry buildings. The outcomes of preceding implementations of SMAs in retrofitting of masonry buildings are then employed to present two comprehensive schemes as well as an implementation algorithm for strengthening masonry structures using SMA-based devices.

## 1. Introduction

Each year, earthquakes cause thousands of causalities and billions of dollars of loss in global property damage [1]. They are caused by the relative displacement of two tectonic plates, which causes deformation of the tectonic plates on both sides of the fault. These deformations persist for several years, accumulating deformation energy in the tectonic plates. Slippage will occur as a result of a malfunction when the plate returns to its original undeformed shape and suddenly releases a large amount of energy. Regardless of the cause of the earthquake, the consequences can be disastrous for human life. Table 1 lists some of the victims of major earthquakes in the past.

The evaluation of the seismic risk of building stock is a challenge for governments and academia in earthquake-prone areas with a high concentration of people and urbanization [2,3]. The natural hazards approach is critical for the establishment of disaster response systems, as well as ensuring disaster preparedness and risk mitigation in accordance with sustainable development [4,5].

Older structures were constructed before earthquake protections were in place [6,7]. Throughout the course of time, many historical structures have undergone various improvements and may have been subjected to calamities such as fires, earthquakes, and explosions [8]. Existing masonry structures can bear both vertical forces (self-weight and applied loads) and horizontal wind forces. However, because most of these structures were not constructed for seismic stresses, they must be reinforced [9,10].

Earthquakes can cause structural and non-structural failures during and/or after earthquakes [11]. Disturbances induced in structural elements of lateral and gravity-load-resisting systems, such as spandrels and piers, and horizontal diaphragms, such as slabs and roofs, are examples of structural failures. Seismic failures in structures are typically caused by either insufficient strength due to inadequate member dimensions, material strength, and seismic resistance or a lack of inelastic deformability. The ability to produce inelastic deformations without significantly reducing strength is known as “ductility”. Brittle construction materials, such as unreinforced masonry (URM), suffer from lack of ductility when exposed to seismic excitations [12,13].

A typical adobe house is a single-story structure with foundations made of medium to large stones and adobe mortar in the middle. The roof is usually made of wooden beams or bamboo, and it is covered with corrugated metal plates, clay bricks, or thatch. In general, the materials used are determined by the region’s economy. As shown in Table 2, adobe structures are prone to collapse during an earthquake, resulting in significant damage and casualties. Furthermore, it is estimated that masonry buildings account for more than 70% of the world’s building inventory [14] and that building collapses were caused by more than 75% of earthquakes in the last century [15].

**Figure 1:**
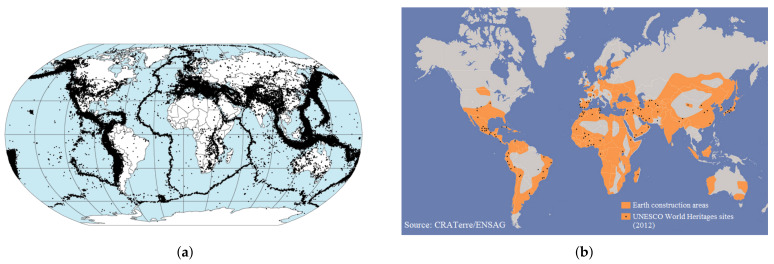
(**a**) Earthquake epicentres in world from 1963 to 1998 (NASA, DTAM project team); (**b**) distribution of earthen constructions (Auroville Earth Institute, UNESCO Chair Earthen Architecture. Buildings with Earth Technique overview, 2012) [22].

Figure 1 depicts and contrasts the global distribution of earthen buildings with the global distribution of seismic hazards. As can be observed, the vast majority of these earthen constructions, as well as world-historical sites, are situated in seismically active locations. Historical heritage sites, which have a variety of characteristics, such as historic, cultural, and psychological, are essential for achieving sustainability in urban conditions [19,20]. As a result, improving the earthquake resistance of these types of structures has been one of the engineers’ top priorities [21].

**Figure 2:**
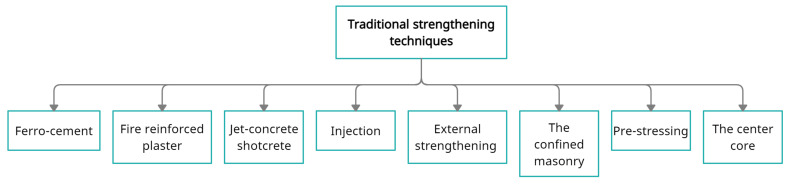
Traditional strengthening techniques of masonry structures [25].

The emergence of wooden elements in the structure of masonry walls in ancient Greece following severe seismic occurrences is the earliest point in history that an attempt was made to establish a systematic technique for seismic resistance to structures [23]. Traditional reinforcing procedures occasionally fail to provide the structure with sufficient resistance to the maximum predicted seismic activity and result in modifications to the original constructive shape that are unacceptable from a cultural viewpoint. Strengthening using these approaches is frequently complicated, leading to disruption of usage, increased financial expenses, and, in some circumstances, unmanageability [24]. Figure 2 also depicts traditional reinforcing procedures for cultural heritage masonry constructions.

One previous destructive earthquake was the Bam earthquake of 2003 that devastated the southern city of Bam, Iran. The epicentre of the earthquake was located at 29.01° N—29.01° E, 10 Km, southwest of the city of Bam. Its magnitude was measured with M_*b*_ of 6.3 and M_*s*_ of 6.5 estimated by the U. S. Geological Survey. This earthquake took the lives of approximately 45,000 people as it occurred at 5:26 a.m. local time when the majority of the population was sleeping. The peak ground acceleration (PGA) at Bam station was 0.8 g and 0.7 g for the east–west horizontal and north–south horizontal components, respectively, and 0.98 g (relatively high acceleration) for the vertical component.

**Figure 3:**
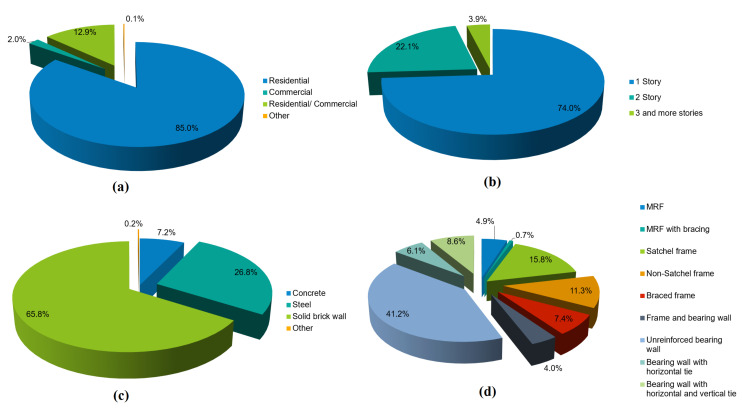
The details of Bam’s buildings prior to earthquake of 2003 [26]; (**a**) types of the buildings’ usage; (**b**) number of stories for buildings; (**c**) distribution of building types; (**d**) structural system types.

Figure 3 shows the share of the buildings’ usage in Bam, the number of floors, the types, and the structural system before the 2003 earthquake. Almost 75% of the buildings inspected were single-story structures with a short lifespan. Masonry buildings with solid brick walls were also by far the most common building type in Bam. Finally, almost half of the buildings used unreinforced load-bearing walls in some form or another, with the satchel type of simple framing being the second most popular system.

In this study, an attempt was made to identify the major causes of the Bam earthquake, taking into account the important influence on the sustainability of masonry constructions that endure seismic pressures during their lifetime. As a result, based on prior research on the use of SMAs in these types of structures, as well as the primary structural failures in the Bam earthquake, two basic retrofitting methods and an algorithm are suggested that may decrease the seismic impacts on URM structures. Such SMA-based retrofitting methods may perform well in general since they may be applied to specific application conditions and regional design codes (as in this paper, the Iranian design code for masonry buildings and earthquake design Standard-2800 are considered).

## 2. Seismic Behaviour of the Structures in Bam Earthquake

The vast majority of buildings in Bam at the time of the earthquake were URM structures with load-bearing walls or low-rise steel-framed structures [27]. The former had either classic masonry domes or vaults or a non-engineered, unanchored jack arch flooring system, whilst the latter had primarily anchored jack arch slabs. Floors in some of the newer structures were composed of concrete beam-hollow block slabs. Weak, fragile materials and element connections, as well as excessive weight, characterize these constructions. The materials and procedures employed in this form of building have remained constant for thousands of years [28].

**Figure 4:**
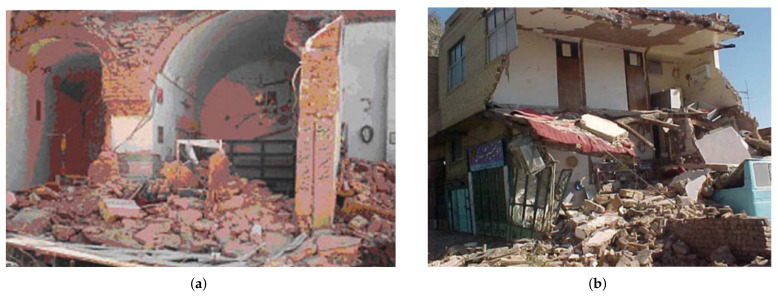
(**a**) Partially damaged old masonry buildings’ arch roofs in Bam after the earthquake [31]; (**b**) the inconsistency of the multi-arched roof with the supports due to the lack of horizontal coils, as well as the high thickness and weight of the roof [31].

Existing structures in Bam were made up of 90% adobe and masonry, 8% steel, and 2% reinforced concrete residential buildings. According to research, 62% of the 550 assessed partially damaged buildings could not be utilized for habitation, 34.8% could be retrofitted, and 3.2% were safe to use [29]. The major reasons for the failure of adobe and masonry buildings, particularly recently erected ones, were heavy roofs and walls, as well as a lack of structural strength. The robust performance of the arch roofs of the old masonry domes demonstrated the need for structural integrity (see Figure 4a). As a result, masonry domes should always be investigated numerically and experimentally in order to obtain enhanced performance maintenance [30].

**Figure 5:**
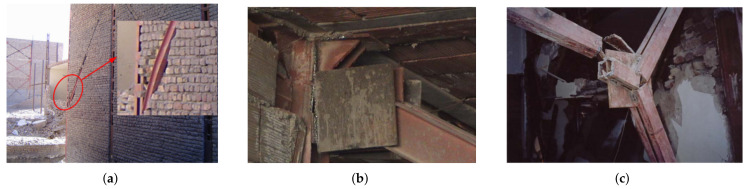
Failure of the lateral bracing system [32]; (**a**) out of plane buckling of bracing system; (**b**) fracture of connection in concentrically X-bracing system due to the poor welding quality; (**c**) bracing failure at splice joint.

**Figure 6:**
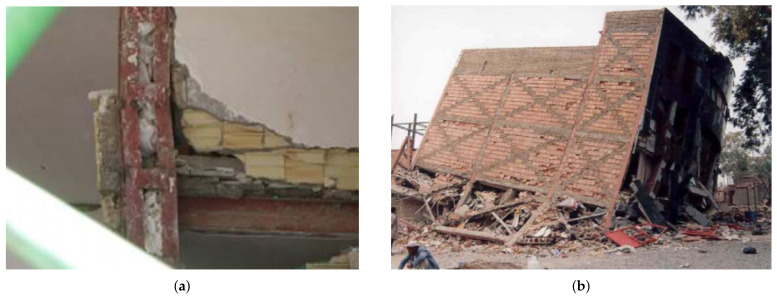
Other types of failures in structural systems [34]; (**a**) buckling of a column in ground floor connected to the balcony beam; (**b**) inappropriate lateral bracing system and longer length of first floor column led to a soft story building.

Most of the steel-framed buildings were damaged due to lack of code implementation, poor workmanship, poor connections, specially satchel connection (Figure 4a), buckling (overall, out of the plane and lateral-torsional as seen in Figure 5a), weld rupture (Figure 5b), local buckling and rupture of X-bracing (Figure 5c), rupture and plastic shear of the battens (Figure 6a), and lack of frame in one direction of the buildings (Figure 6b).

The battens are used to distribute shear between the chords of a column. As a result, failure of battens results in chord separation and a significant reduction in the axial capacity of a batten column. The magnitude of the modified slenderness ratio of the column about a hollow axis grows as the distance between the battens grows, and therefore the axial capacity of the column may decrease or change the direction of buckling.

Overall buckling, local buckling of one chord, lateral-torsional buckling, and batten failure are all dominant failure mechanisms of batten columns. Some of these types are caused by a significant decrease in the axial capacity of the batten column and, as a result, must be avoided [33].

**Figure 7:**
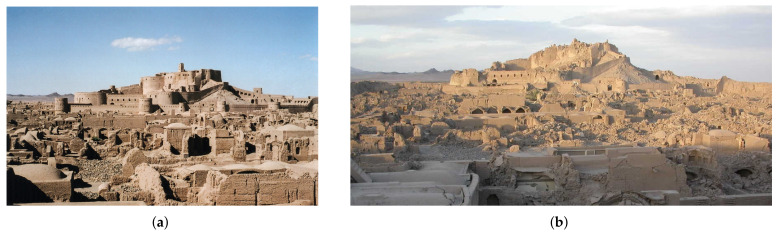
Bam citadel; (**a**) before the 2003 Earthquake; (**b**) after the earthquake (UNESCO).

The Bam Citadel was an outstanding example of earthen construction that was severely destroyed in the 2003 Bam earthquake as displayed in Figure 7. Up to 95% of the structures and walls of the 2500-year-old historic fortress, the world’s biggest adobe edifice, were destroyed. The failure was mostly caused by inadequate seismic safety considerations in the repair work.

According to research [35], it is assumed that the collapse of the Citadel’s walls was caused mostly by a combination of the following effects:

- The additional alterations to the walls, especially in recent restorations, that resulted in differences in the density and reactivity to vibrations of different layers of unloaded earth construction in the walls;
- Extensive termite infestation, as well as loss of clay cohesion due to deterioration and excessive drying out, all combined with the extremely high-frequency earthquake vibrations in such a way that many walls virtually broke due to the loss of cohesion and sinking of their clay interior cores.

From another perspective, the authenticity and truth of historical structures and heritage sites [36] can be easily compromised and that is why they have to be thoroughly studied from different aspects for proper management and maintenance [37].

The original impression was that the twentieth-century restoration work functioned significantly worse than the ancient work; nevertheless, the newer work frequently collapsed as a result of the internal collapse of the older work on which it was based. Internal wall degradation-dryness and lack of cohesiveness of the earthen cores, decay and consumption of reinforcing timbers and fibre reinforcements, and the presence of tiny and large holes between vertical layers in the walls all could have played a role in the collapses [35].

After reviewing the previous studies that investigated the main causes of the collapse and deflection of the buildings as well as the Bam citadel, a study [38] introduced the main major deficiencies of the Bam earthquake, outlined as:

- Inadequate connections between walls and roofing in masonry and steel-framed buildings;
- The vaulted ceiling lacks consistency;
- Weak portions around the dome’s junction line and the residual flat part of the roof;
- High weight of the roof;
- Inadequate retrofitting of the historical heritage;
- Poor quality in manpower and construction work;
- Inadequate design parameters;
- Lack of using flexible and high-strength reinforcing materials.

In general, one of the most important lessons that structural engineers should learn from the Bam 2003 earthquake is considering the significance of retrofitting the buildings and historical heritages. The collapse of the Bam citadel and its consequences proved that retrofitting a historical structure may profit from the use of novel materials with great strength, self-healing capacity, strong re-centring capabilities, etc. Materials with such characteristics can effectively moderate the input seismic energies and prevent the structure from being damaged or collapsing. Shape memory alloys (SMAs) are an appealing class of metallic alloys that have attracted scientists for decades. They can be utilized as reinforcing materials in retrofitting existing buildings to strengthen the structures against earthquakes. The next sections describe the fundamental properties and behaviour of these groups of alloys, as well as their uses in masonry structure retrofitting.

## 3. Basics of Shape Memory Alloys

Metals have long been regarded as pillar building materials for civil constructions. Shape memory alloys (SMAs) are a class of metallic materials that have the potential to revert to their original shape after being subjected to large deformations (strains of 8–10%). This shape recovery is a result of the martensitic phase transformation induced by either heating (known as shape memory effect) or removal of the applied stress known as pseudoelasticity (or superelasticity). The first discovery of the shape memory effect (SME) was recorded in 1932 by Ölander [39], although the term “shape-memory” was first introduced by Vernon in 1941 [40]. Since the 1960s, after the discovery of Nitinol by Buehler [41], SMAs have drawn considerable attention from investigators in different research fields including metallurgy, crystallography, mechanics, and mathematics [42,43,44,45,46]. Applications of SMAs in civil and structural engineering have been developed for about three decades now [47,48].

**Figure 8:**
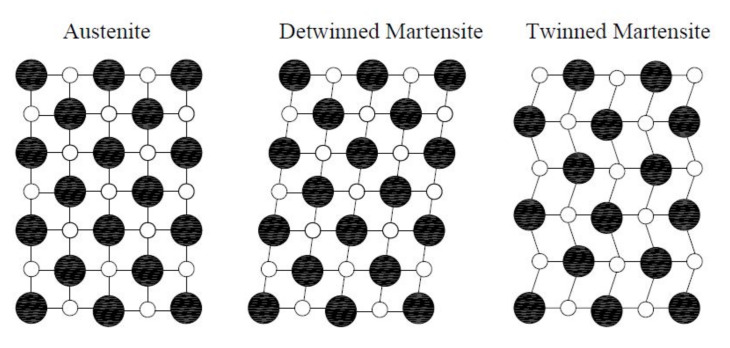
Different crystal structures (phase structures) of SMAs.

**Figure 9:**
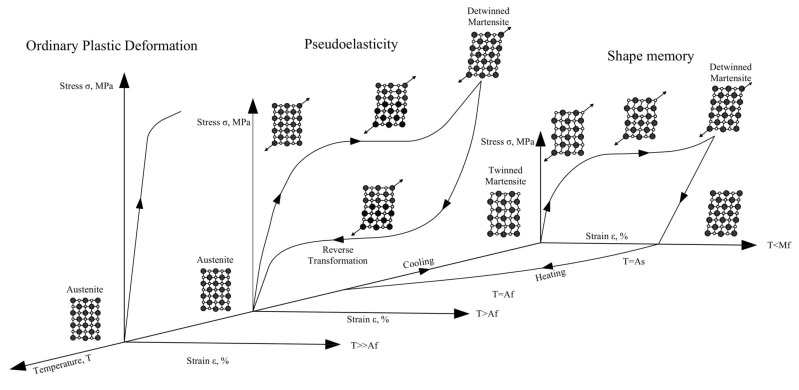
Stress–strain diagram of SMAs and the schematic crystal structures at different temperatures [54].

SMAs with the same atomic composition can exist in several crystal structures as shown in Figure 8. The austenite stronger structure is stable at higher temperatures and lower stresses, and it is also known as the cubic crystal parent phase. Martensite is more stable at lower temperatures and higher stresses due to its crystal structure with a lower level of order. The unique properties of SMAs, which result from the reversible martensitic phase transformations, are illustrated in Figure 9. Modelling and numerical simulations of boundary value problems for SMA structural elements represent a real challenge due to the dissipative nature of the deformation process of reversible phase transformations that constitute a non-convex problem with hysteresis loops [49,50,51,52,53].

The transformation from martensite to austenite can develop in the absence of applied stresses just by heating an SMA. The austenite commencing temperature ($A_{s}$) is the temperature at which the material changes from twinned martensite to austenite. The temperature at which this change is complete and the material totally converts into austenite is referred to as the austenite finish temperature ($A_{f}$). In other words, as the SMA’s temperature rises above a certain threshold, its initial shape converts into the austenite structure. This transformation phase can occur even with large applied forces, resulting in high actuation energy densities. Austenite turns into twinned martensite throughout the cooling process at the martensite start temperature ($M_{s}$), and the transition completes at the martensite completion temperature ($M_{f}$). Variations in the relative quantities of the component metals, the manufacturing process, and applied stress to an SMA can all affect the transformation temperatures. The greatest temperature at which martensite can no longer be stress-induced and beyond which the SMA is permanently distorted like any other regular metallic material is referred to as $M_{d}$ ($M_{d}\gg A_{f}$).

Thanks to the excellent properties of SMAs including good hysteretic behaviour, great re-centring capability, high strength, high fatigue and corrosion resistance, and substantial damping capacity, SMA-based structural components may be used in a wide range of applications in civil constructions. Seismic-resistant design and retrofitting of existing buildings and infrastructures are some of the areas where SMAs have feasible potential.

Up to this point, among the many SMAs, Ni-Ti alloy (also known as Nitinol) has demonstrated exceptional shape memory behaviour, with large magnitudes of shape recovery, recovery stress, and super elastic strain. As a result, Ni-Ti offers a wide range of applications, including structural reinforcement, vibration building control, and structural self-repair. For the last decades, binary Ni-Ti alloys, as well as ternary Ni-Ti–X alloys (X stands for potential supplementary alloying components), have been the most appealing types of SMAs.

However, for many years, the exorbitant costs of manufacturing and deploying Ni-Ti SMAs in buildings have been a deterrent to their widespread use. As a result, there is a rising attempt to employ more cost-effective SMAs, such as Cu- and Fe-based ones, in order to achieve a wider spectrum of utilization.

Because of their outstanding mechanical capabilities, Fe-SMAs are suitable for usage as reinforcing materials, such as in reinforcing existing civil structures and prestressing new civil structures. Most iron-based SMA compositions in bulk are Fe, which is an inexpensive and easily accessible material. As a result, they are a low-cost category of SMAs that exhibit SME and high ductility [55].

To demonstrate the importance of utilising SMAs in masonry building retrofitting, the characteristics of Ni-Ti and Fe-based SMAs are compared to stainless steel in Table 3. Other new retrofitting approaches, such as utilising FRP materials and steel jackets, have the following drawbacks:

- The steel jacketing approach has certain drawbacks, including the ability to rust and the difficulty of installing by machinery. The grouted area, which is the gap between the concrete void and the steel jacket, clogs up, resulting in a column inconsistency [56].
- FRP materials have a low elastic modulus (10 times that of steel), lack of ductility, and inferior shear strength [57].

Compared to other aforementioned novel retrofitting approaches, thanks to their superior properties, SMAs are quite desirable in masonry retrofitting applications. It becomes apparent that, in general, the use of SMAs would enhance the construction owing to their excellent properties when compared to traditional reinforcing materials such as steel.

## 4. Shape Memory Alloys Application in Masonry Structures and Historical Buildings

The degradation of historical building materials, extended exposure to climatic factors, and uneven settlements require the restoration of historic structures. Furthermore, many ancient buildings were designed to withstand gravity loads primarily or at a significantly lower seismic intensity than comparable modern ones. Seismic retrofit procedures must then be integrated into the structure without affecting its appearance. However, necessary structural elements for absorbing the out-of-plane horizontal thrusts generated by arches, vaults, and wooden roof trusses are often lacking, insufficient, or degraded [58].

In general, the seismic retrofitting techniques for masonry structures can be classified as follows:

- Reducing the earthquake forces that could be exerted on the structure;
- Improving the existing building to resist earthquake load through the change in the structural system or enhancing the elements’ strength.

To retrofit existing structures, it is necessary to first assess the building’s seismic performance and, as a result, to determine the causes of inadequate seismic capacity. Based on this assessment, an appropriate retrofitting technique might then be designed [59]. A successful retrofitting solution must take into account the sustainability factors (technical, economic, and social). In addition to these considerations, the environmental effects of an earthquake should be considered since they have a substantial impact on the global ecosystem, and structural optimization might efficiently decrease carbon emissions by constructions [60]. Each retrofitting methodology has advantages and downsides; a technique that is good for one structure may not be appropriate for another. The chosen method must be compatible with aesthetics, function, strength, ductility, and stiffness, as well as cost constraints.

In addition to their unique re-centring ability and significant energy-dissipating capacity, superelastic SMAs also have favourable properties such as the ability to endure large deformations, good fatigue, and corrosion resistance. In this section, we first attempted to trace the historical background and introduce the most important developments and innovative structural response control systems in the seismic applications of SMAs. This is followed by the seismic retrofitting of masonry buildings by the implementation of SMA-based devices.

Up to now, many researchers have investigated the application of SMAs in seismic control systems. The application of SMAs in the seismic control of structures can be categorized into different groups such as base isolation devices, passive and active vibration controls, etc., as listed below:

- Vibration control systems;
- Base isolation devices;
- Energy dissipation devices;
- Active vibration control;
- Semi-active vibration control.

In this study, some masonry building cases, with deficiencies similar to those in the Bam earthquake, that have been retrofitted by using SMAs are reviewed. The authors are aware that in every retrofitting process, comprehensive studies and designs have to be done related to the local codes and the seismicity of the region to achieve high-post-retrofit structural performance while undergoing seismic forces. However, according to the authors’ awareness, the expanding research and use of SMAs for civil constructions might anticipate that, in the near future, these materials will be able to play a large part in retrofitting masonry buildings worldwide, alongside other technologies such as the use of FRPs. That is why this evaluation focuses on prior successful applications in order to help and enhance future research.

### 4.1. Experimental Investigations

#### 4.1.1. Experimental Investigation of Retrofitted Half-Brick Walls with Cu-Al-Mn SMA Bars

Shrestha et al. [61], by performing quasi-static tests on half-scale brick walls subjected to cyclic out-of-plane flexure, evaluate the suitability of Cu-Al-Mn SMA bars to the retrofitting of ancient masonry projects. The experimental and computational findings revealed that both the steel-reinforced and SMA reinforced examples had a significant increase in strength and durability when compared to the unreinforced sample. The steel reinforcement in the strengthened sample experienced compression and significant residual deformation, while the superelastic characteristic of SMA bars resulted in a rather steady reaction with no compression phenomena. Both experimental and computational results show that Cu-Al-Mn SMA bars outperform conventional steel reinforcement in retrofitting old masonry buildings.

This study demonstrated the advantages of SMA bar retrofitting of URM walls in a more realistic context, and its results could be summarized as the following:

- When compared to the URM specimen, both the steel-based and SMA-based specimens demonstrated considerable increases in strength and ductility. The steel-based specimen experienced pinching in the moderate displacement range, but the SMA-based specimen exhibited nothing. These findings illustrate the suitability and effectiveness of the current Cu-Al-Mn SMA bars as a partial substitution for steel bars in retrofitting URM walls.
- To reproduce the experimental data, finite element (FE) models were created and evaluated. The created FE models identified the whole history of all the specimens quite accurately. The inelastic elongation of the steel bars was found to be the major cause of pinching in the FE study of the steel-based specimen. It was also demonstrated that the superelastic feature of the SMA bars was beneficial in preventing pinching.
- When reinforcing bars were installed at the underside of the wall specimen, the steel-based model displayed pinching even in the minor deformation range, while the SMA-based model did not exhibit such deformation.

#### 4.1.2. Experimental Investigations on Pre-Tensioned SMA Wires on URM Walls

**Figure 10:**
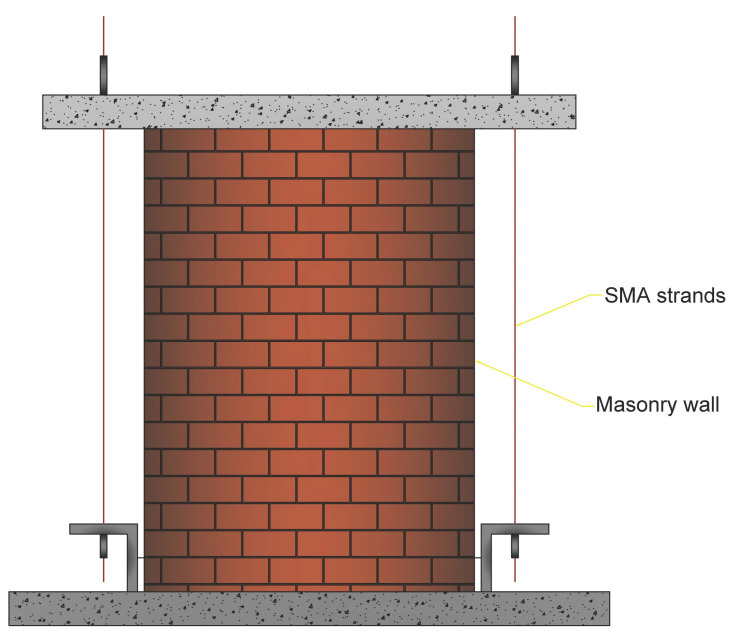
The schematic of the proposed system with SMA wire for connection ties [62].

Casciati and Hamdaoui [62] investigated the use of pre-tensioned SMA wires to retrofit historic masonry structures. They evaluated the structural aspects associated with the use of an SMA-based connection ties system as shown in Figure 10.

The findings of laboratory studies were used to construct a numerical model that includes the impacts of the SMA devices. The structure was first examined in its original condition, without any retrofitting attempts, and then the impacts of various retrofitting approaches were empirically tested and integrated into the numerical model by modifying the equivalent Young’s modulus correspondingly.

**Figure 11:**
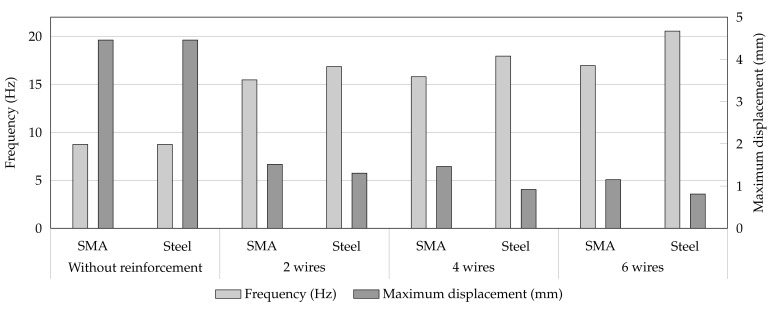
The effect of using SMA or steel with different number of wires on frequencies and maximum displacement of the wall [62].

They compared different engineering factors between the usage of SMA and steel wires with the different number of used wires in the retrofitting of masonry wall under 20% of the El-Centro earthquake, which is demonstrated in Figure 11. These factors include frequencies and maximum displacement in systems without and with a different number of reinforcement (SMA or steel-based) wires. Furthermore, the equivalent modulus of elasticity (since Young’s moduli of the system’s elements are different, an equivalent value is elaborated) of the system without any wire was equal to 5875 MPa, whereas this value was recorded as 20,665, 19,180, and 22,100 MPa for the systems with 2, 4, and 6 SMA wires, respectively.

The authors of this study introduced three main advantages of using ties of SMAs in the austenitic phase in the retrofitting of masonry buildings:

- By requiring the prescribed pre-tension to reach the super-elastic constitutive law plateau, no extra stress is transmitted to the masonry at higher strain levels. As a result, no springs are required to be included in the computational model, with the potential of mutual deformations between the clay bricks, i.e., energy absorption.
- The hysteresis loop of the hyper-elastic stress–strain relation dissipates more energy.
- The ties may re-centre themselves in their original location with no residual displacement.

### 4.2. Numerical Modelling of Retrofitted Structures with SMA Devices

#### 4.2.1. Post-Tensioned Iron-Based SMA Strips in Retrofitting of URM Walls

**Figure 12:**
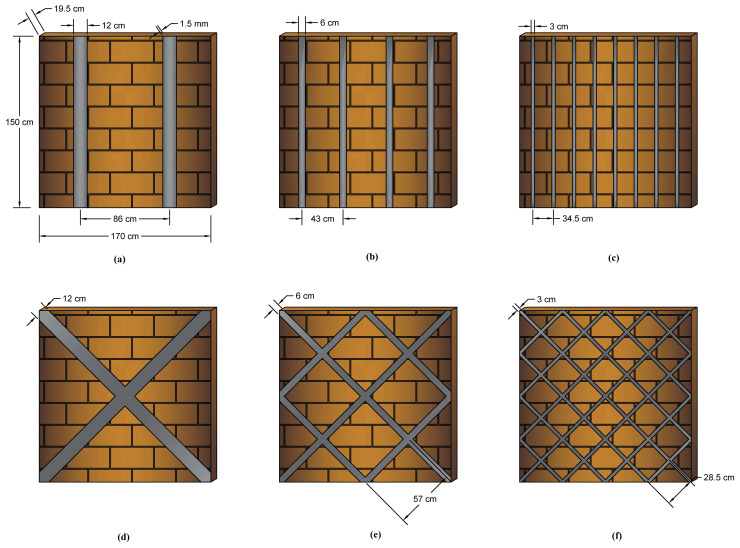
Numerically studied SMA-reinforced walls [63]; (**a**) 2 vertical stripes; (**b**) 4 vertical stripes; (**c**) 8 vertical stripes; (**d**) 2 cross-strips; (**e**) 6 cross-strips; (**f**) 14 cross-strips.

Rezapour et al. [63] analysed the performance of URM reinforced with iron-based SMA strips. As seen in Figure 12, the SMA-based strips were mounted in the shape of crossings and verticals in walls and subjected to post-tension force with mortar as the connector between the wall and strips.

The results of this study demonstrated that in the vertical-strip walls, the stiffness increased by 98.1%, and in the cross-strip model’s position, the stiffness increased by 127.9%. Furthermore, in the vertical-strip model, the maximum resistance was equal to 108 kN, while in the end cycle, it reduced by almost half and reached 40 kN. In large deformations, the greater the scattering of the iron-based strips, the more resistant the masonry wall is. Thus, in the model in Figure 12f, the maximum resistance is equal to 104 kN, and in the final cycle, this number decreases by only 13.5% and reaches 90 kN.

As seen in Table 4, the walls with SMA cross-strips dissipate more energy than the other models. The strength in cross-strip models is larger than in other types, particularly in the final cycles, which enhances the energy dissipation efficiency. Since the model in Figure 12c had the greatest energy dissipation between other vertical-strip systems, the authors concluded that the greater the dispersion of SMA strips on the brick wall, the greater the energy dissipation.

The quantity of energy dissipated during the cyclic loading is shown in Table 4. As that brick wall’s behaviour was almost linear in the initial cycles, the energy loss values were negligible (about zero) up to six cycles. The proportion of dissipation of energy rises when a plastic deformation is applied to the system.

#### 4.2.2. SMA Cables in Seismic Protection of a Historic Brickwork Church in Northern Italy

**Figure 13:**
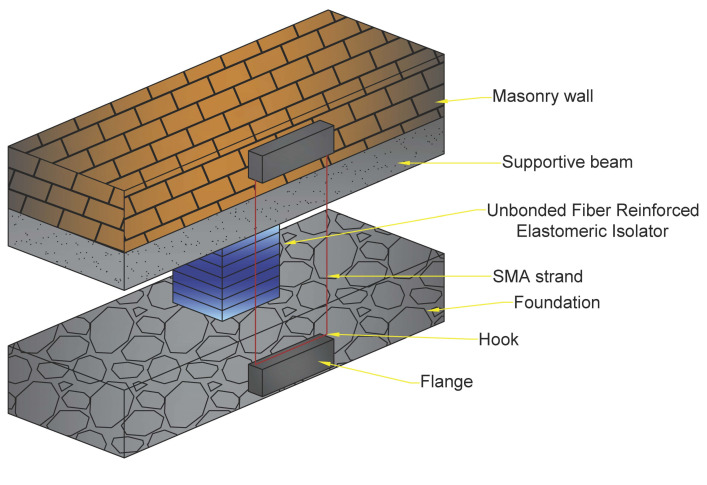
The schematic of the proposed system and SMA strand in the isolation system of masonry constructions [64].

Habieb et al. [64] studied the use of an embedded base isolation system for seismic protection of a historic brickwork church. The system was built with an unbonded fibre reinforced rubber isolator and SMA cables (see Figure 13).

Due to its high energy absorption capacity, the suggested model with a 2% pre-strain SMA wire model demonstrates the biggest reduction of the lateral deflections of the church and significantly lowers impact (from destruction to mild damage level) in the event of powerful earthquakes. It should be mentioned that the model without pre-strain in the SMA strand is less efficient than the pre-strained ones in decreasing damage and lateral deformations of the structure. Finally, even for high PGA, the global seismic response of the isolated church is not significantly different when comparing the usage of a pre-strained-SMA-based system with the model without SMA wires. On the other hand, in the case of SMA application, the fair decrease of isolator displacements, particularly under high PGA, may be regarded as an essential advantage because unbonded isolators are susceptible to sliding destabilization when subjected to substantial deflections.

In the case of PGA = 0.3 g, the use of SMA provides evident advantages. In reality, in the model without SMA wires, substantial residual vibrations occur towards the conclusion of the earthquake stimulation and progressively dissipate. The other two models (pre-strained and non-pre-strained SMA wires) exhibit minimal residual vibrations at the conclusion of the earthquake excitation due to the SMA wires’ superior damping and energy dissipation capacity at large deformations.

It is worth noting that practically all of the fixed-base model’s reference points exhibit drifts greater than the limit value associated with the collapse level. The base-isolated models greatly minimize the displacements from failure to levels of minor/moderate damage. Among the base-isolated models, the pre-strained-SMA-based model had the greatest reduction in deflections. The non-pre-strained-SMA-based model, on the other hand, exhibits relatively high residual displacements. Such findings indicate that the use of SMA wires in an elastomeric isolation system is more beneficial in the event of large earthquake excitation, with a specific degree of pre-strain used to keep the isolation system rigidity moderate.

### 4.3. Implementation on Real Structures

#### 4.3.1. SMA-Based Device in Retrofitting of San Paolo Eremita Church in Southern Italy

**Figure 14:**
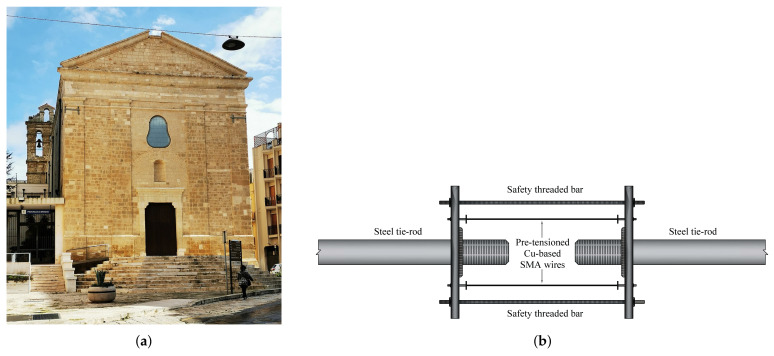
Retrofitting of the San Paolo Eremita church; (**a**) San Paolo Eremita church (photo by Saggittarius A.) (**b**) schematic of the proposed SMA-based system [58].

Cardone et al. [58] developed a proposed system based on the superelasticity features of copper-based SMA to improve the thermal and seismic behaviour of steel connectors in historical structures (San Paolo Eremita church (see Figure 14a)). Experimental results show that the suggested device is effective at reducing force changes caused by changes in air temperature. The testing findings clearly demonstrate the proposed SMA-based device’s high efficacy in improving the thermal behaviour of steel connectors. Indeed, with SMAs, the force fluctuations in the steel tie-rods caused by air temperature fluctuations are 80–90% smaller than those without SMAs. Shaking table experiments reveal that using the suggested SMA device can improve the seismic performance of buildings mounted with alloy tie-rods. This is mostly due to the SMA device’s increased energy dissipation capabilities and re-centring ability. For facilities in high seismicity territories where minor temperature variations are predicted, ideal seismic behaviour of the SMA unit should be addressed; for structures in low seismicity zones where significant temperature fluctuations are predicted, the ideal temperature behaviour of the SMA unit should be followed.

The suggested model, seen in Figure 14b, links the two steel tie-rods with pre-tensioned copper-based SMA strands. The hysteretic performance of the connector with the SMA device during the tests with the artificial ground motions at 0.6 g was compatible with the strain limit of SMA (equal to 9–10%, equivalent to the end of the martensitic transformation) and yield force of the steel tie-rod, equivalent to approximately 3 KN. Furthermore, the system equipped with the SMA unit exhibited excellent re-centring capability. The tie-rod without an SMA device, on the other hand, had severe plastic deformations with considerable residual displacements of the magnitude of 21 mm at the end of the earthquake, equating to a residual deformation in the steel rod of a scale of 2.4%. During testing on the structure with an SMA unit, no destabilizing behaviours were detected, however bending of the steel bar was detected frequently during the testing on the structure without an SMA unit. When the tie-rod backs in tension, it causes shocks to the structure around the anchorage plates.

Another aspect that deserves further studies concerns the opportunity of using virgin wires or wires trained by a few loading cycles before installation. Indeed, shaking table tests have shown that the use of virgin SMA wire is desirable. Two distinct techniques were considered during the implementation. Both ideas were fully achieved, demonstrating the viability of both options. Additional devices were installed and monitored over time to give more information and ideas to improve the installation method.

#### 4.3.2. SMA-Based Device in the Restoration of the Bell Tower of San Giorgio at Trignano

**Figure 15:**
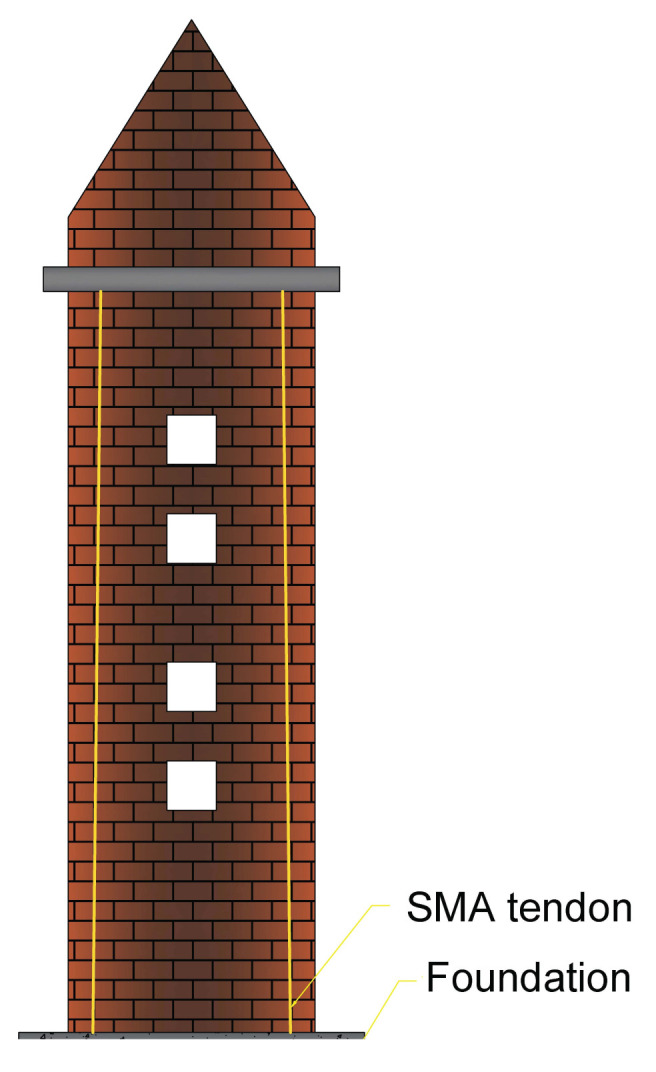
Installation of four pre-tensioned steel tie bars and SMA devices in the interior corners of the S. Giorgio church bell tower that were anchored to the foundation and the roof [65].

Castellano et al. [65] investigated the application of SMA in historical heritage seismic retrofitting. They used the SMA-based device in the restoration of the Bell Tower of San Giorgio at Trignano with four pre-tensioned SMA devices at the corners of the tower (see Figure 15). The practicality of employing SMA devices with varying behaviours was proved by the development of a number of prototypes that were extensively tested. Shaking table studies revealed that a new binding approach based on SMA devices can be extremely successful in preventing the out-of-plane collapse of outer masonry walls, church façades, and insufficiently linked at ground floor structures.

It was observed that the SMA device exhibits good acceleration reduction: for example, almost 50% at the top and more than 60% at the connections level. Furthermore, the maximum force peak in the wall reinforced with the SMA device is reduced by 45%. When compared to standard ties, SMA device ties can boost resistance against out-of-plane seismic vibrations of such masonry walls by at least 50% (in terms of maximum PGA acceptable without degradation), contributing to a 50% reduction in peak acceleration. Furthermore, unlike standard steel ties, SMA device ties may avoid tympanum structural collapse. Pseudo-dynamic, in-plane experiments on masonry wall mock-ups with openings revealed that SMA devices used in conjunction with steel tendons to prestress the masonry may absorb around 30% of the seismic input energy and thereby increase a structure’s seismic resistance.

## 5. Retrofit Techniques with SMA-Based Device for Masonry and Adobe Buildings

After reviewing different case studies that examined and determined the efficiency of the application of SMAs in URM buildings, this section provides the schemes of URM structural elements retrofitted with SMA-based components. The schemes are based on the Iranian national design codes for masonry buildings in accordance with the most common failure modes in the Bam 2003 earthquake. The collapse of the walls and lack of strength in lateral resistance frames are considered two of the most common reasons for the damages to the buildings in the Bam earthquake. Generally, the most common failure modes of masonry buildings are listed as follows [66]:

- Lack of anchorage;
- Anchor failure;
- In-plane failures;
- Out-of-plane failure;
- Combined in-plane and out-of-plane effects;
- Diaphragm-related failures.

**Figure 16:**
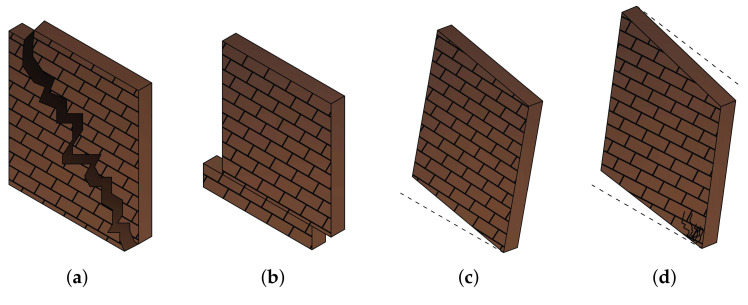
In-plane failure modes of URM walls; (**a**) shear failure; (**b**) sliding failure; (**c**) rocking failure; (**d**) toe crushing.

Additionally, as for masonry walls, since connections between neighbouring walls are sufficiently strong, the wall’s in-plane shear resistance is deployed, and shear fractures form. Once a building is fractured since there is no further connection between the neighbouring components, the dynamic reaction is similar to rocking instability [67]. Besides rocking, Figure 16 depicts other possibilities for the in-plane failure of masonry walls.

Shadbin et al. [68] provided the results of a shaking table test of a single-storey URM building with a Jack-arch ceiling as a proper representative of existing URM buildings in Iran, which also was one of the most common structural types in Bam before the earthquake of 2003. Experimental results revealed that the recommended strengthening system in Standard-2800 [69] for the roof diaphragm in jack-arch slabs was efficient as an effective solution for reducing the seismic vulnerability of existing masonry buildings with flexible floors.

Furthermore, based on the latest version of the Iranian design codes for masonry buildings [70] and seismic design standards [71], the implementation of steel reinforcement in masonry walls needs to be followed by the undermentioned regulations; however, on the conceptual level, these rules can be applied for further applications of SMAs as reinforcement materials in URM walls:

- The rebars must be entirely encased by building materials (for example, mortar), and the strain connection between the mortar and the rebar must be such that the applied loads are carried in a compound way.
- Vertical bars with a minimum value of 130 mm^2^ should be placed at each junction of two or more walls, as well as at the ends of the walls. In addition, for the length of the wall, at least 130 mm^2^ of vertical rebars shall be inserted, with a maximum horizontal spacing of 1200 mm (across the wall).
- A minimum horizontal bar with a cross-sectional area of 130 mm^2^ should be considered for each of the following: above the wall and at the point of continuous connection of the ceiling or floor to the wall; at the bottom of the wall or above the foundations if the foundations are attached to the walls; concentrated at intervals of up to 3 meters or uniform in height.
- The minimum yield strength of the rebars (*f_y_*) and the minimum characteristic strength of concrete (*f_c_*) must be equal to 240 and 20 MPa, respectively.
- Using rebars with a diameter smaller than 10 mm is not allowed.

Up to now, there has not been any consideration of the application of SMAs in the Iranian design codes, which is why prior to any implementation of SMA-based devices, further investigation is required to obtain sufficient seismic resistance.

**Figure 17:**
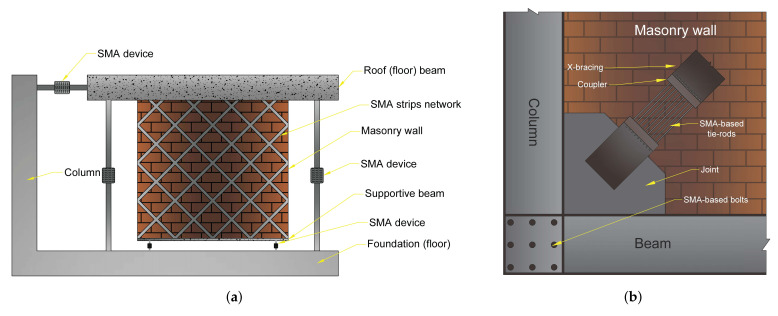
(**a**) Schematic of a retrofitted masonry wall by the application of SMA devices; (**b**) retrofitting of the beam-column connection and the X-bracing system of the steel frames in masonry buildings.

Figure 17a demonstrates the general schematic of the retrofitting of URM walls by SMA-based devices and/or components considering aforementioned studies, Standard-2800, the Iranian national code for masonry buildings, and the most common failure modes of the Bam earthquake. An SMA device is designed between the roof and its neighbouring column to provide integrity at the conjunction. It also might reduce the maximum deflection of the floor due to the high energy dissipation capacity and re-centring capacity of SMAs. Between the masonry wall and the foundation, a lateral SMA-based resisting system is located, aiming to prevent the four mentioned failure modes of URM walls (Figure 16) and the soft-story building. Furthermore, the SMA device between the floor and foundation might assist in mitigating the earthquake energies and diminishing the level of damage to the wall.

The SMA strips network, which can improve the performance of the URM walls, is employed in order to enhance the strength, stiffness, and energy dissipation capacity of the wall during an earthquake. As the crossing network has shown better behaviour [63], in this scheme, the same pattern is proposed. The network might be helpful to prevent shear, sliding, and rocking failure of the URM wall. Additionally, the supportive beam underneath the wall is designed to prevent toe-crushing of the masonry walls.

In addition to the failure of URM walls, the other significant failure modes of the buildings at the Bam earthquake were the insufficient performance of bracing systems and the weak connection in beam-column joints, mostly out-of-plane buckling and rupture of the connections. By effective dissipation of the energy exposed to the bracing system, the lateral resisting systems could be sufficiently utilized during an earthquake. In the event of deforming the frame structures under excitation, SMA tie-rods dissipate energy through stress-induced martensite transformation and prevent buckling in the bracing elements.

Figure 17b represents a general conceptualization of the connection between the X-bracing element to a beam-column joint. The beam-column connection strengthened with SMA bolts benefits from the high energy dissipation and re-centring capacity, providing high ductility and rigidity. The vulnerability of the joints to external disturbances reduces due to the greater dissipated energy of SMAs by large plastic deformations and then recovering to their original shape [72,73].

These two schematic concepts are provided at a general level, which might be adjusted and then utilized according to regional regulations and required design parameters. The authors’ main idea was to provide such a conceptualized scheme that might be deployed based on different parameters which can affect the shape, dimensions, material’s properties, etc. However, in the authors’ future studies, the performance of these two schemes will be evaluated by different parameters to provide a more specific scheme responding to specific situations.

In each of these retrofitting schemes, some practices have to be fulfilled to obtain the expected outcomes. These practices and results are demonstrated in Table 5.

**Figure 18:**
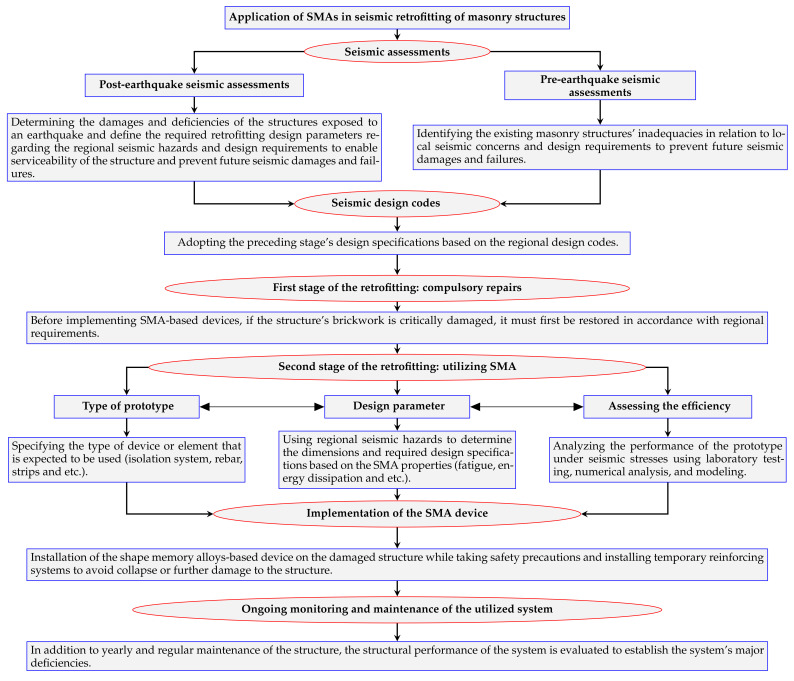
Proposed algorithm of retrofitting of the masonry structures with implementation of SMA-based devices.

After evaluating the methodology and the results of the previous research, as well as the two proposed retrofitting schemes, a generic algorithm is presented that depicts the procedures for integrating SMA devices in masonry structures, a strategy to reduce the seismic risk of masonry structures. Figure 18 depicts the six major steps of this method.

Inspections and seismic evaluations should be used to determine structural deficiencies in the initial phase. This step may include evaluating structures after an earthquake to repair and retrofit a damaged structure or identifying the inadequacies of a constructed structure in order to prevent future seismic failures. Then, in the following step, an optimal seismic design should be supplied for future retrofitting techniques based on local construction codes. Depending on the damaged grade of a structure, some pre-implementation repairs may be required in the first phase of retrofitting; for example, if a URM wall is critically damaged and is unable to provide sufficient strength (to withstand dead and/or live loads), it must first be repaired before any further seismic retrofitting.

The implementation of SMA-based devices consists of three interconnected sub-phases. The type of the desired device should be defined in terms of its utilizing region; for example, if the retrofitting is aimed at improving the isolation between the wall and foundation, the SMA device should correspond to the requirements of such an application. Following the determination of the device’s kind, its design parameters should be developed in accordance with earthquake design rules. Since SMAs are relatively new materials, they are not commonly included in design codes; hence, before any implementation, the performance of the described system should be evaluated in another sub-phase. As a result, these three sub-phases are co-related in this method. If the proposed device’s performance evaluation is insufficient, altering the design parameters and/or device type may result in a satisfactory implementation.

It is essential to note that prior to installing the SMA devices, the structure must be secured to avoid possible failures, collapse, and harm to the people and machinery engaged in the retrofitting operation. For example, in order to install a post-tensioned SMA device, a temporary resisting system must be provided to withstand the forces before the SMA device can be completely employed. The final stage includes the maintenance of the structure and the employed device and monitoring the performance of the system to determine the probable required repairs to reduce the risks caused by seismic events during the life cycle of a structure.

## 6. Conclusions and Discussion

Despite its enormous fatalities and losses, the Bam earthquake tragedy presents a rare opportunity to raise world awareness of the significance of efficient execution of a comprehensive earthquake risk reduction program in Iran as well as other hazard-prone developing nations. It challenges governments to make the most use of current earthquake knowledge and integrate it into development plans.

The major structural factors of the Bam earthquake may be divided into two categories: first, the damages to buildings and infrastructures resulted in a large number of casualties and difficult post-seismic developments; second, the collapse of an old-world heritage site (Bam Citadel). All of these concerns might be effectively avoided by a well-planned retrofitting effort for the historical section, as well as improved building inspections and national regulations.

The use of SMA-based devices to strengthen earthen and masonry constructions is one of the creative methods that might be applied. Based on a study of prior research, using SMA devices can effectively moderate seismic forces and prevent structural damage and collapse. SMAs benefit from the shape recovery functionality, which may also be employed for post-earthquake repairs, which speeds up the repair process and saves time and money. Another attribute of SMAs is their ability to withstand large deformations without rupture, which masonry buildings do not have. In general, the use of SMAs in masonry structures has shown to be an effective technique to protect ancient structures as well as thousands of lives. This study is part of a larger research program examining the use of SMAs in structural retrofitting. Future research will look into the use of SMA devices in the Bam Citadel to prevent it from collapse again following its retrofitting phase.

Additionally, it is important to consider the reversibility of any further intervention in historical structures. Cultural heritage sites are excellent illustrations of how heritage is rarely an end artefact, but rather a continuous cultural product that is constantly being moulded and altered. In this regard, any previous intervention, as well as any contemporary intervention, is irreversible and will influence how present and subsequent descendants perceive the heritage [77]. It is critical to protect the integrity of interventions while simultaneously using performance enhancement to increase the efficiency of historic site maintenance. The intricacy of interventions on historic sites and buildings necessitates a unique strategy that takes into consideration both the intricacy of existing historic structures and the uniqueness of historic construction materials and techniques [78].

There are two requirements for maintaining these objectives [79]:

- Preserving the historical artefact’s construction rationale;
- Utilising materials and processes that are reversible.

Furthermore, marginal technological interventions should be considered, i.e., interfering more than enough to enable an object to maintain a condition of usability, but as little as possible to prevent excessive substitution of historic fabric, thus further guaranteeing the ethics of integration and reversibility in each interference [80].

The authors presented conceptual refurbishment strategies for achieving the optimal seismic effect on a masonry structure during an earthquake. The method is based on extensive literature research and national codes, and it will be further expanded and explored by the authors in various case studies, notably for historical masonry structures, which are seen as a major concern. Overall, this system might help construction engineers achieve a building design and plan to reduce seismic risks, which might lead to long-lasting structures with minimal earthquake damage. Finally, this article is associated with a broader research plan aimed at improving structural performance under seismic forces through the use of SMAs.

In this investigation, the authors focused on papers available in scientific databases and publications. The given cases are based on findings obtained by researchers all across the world. The authors are concerned that local practices and regional design codes may impact the outcomes of utilizing the suggested methodology, but at a conceptual level, the technique should be beneficial and fulfil its goal in terms of local practices, which will be explored and evaluated further in the future.

## Figures and Tables

**Table 1:** Earthquakes with the highest number of casualties in the last century.

Year	Location	Fatalities
1908	Messina, Italy	70,000–100,000
1920	Gansu, China	200,000
1923	Kanto, Japan	143,000
1927	Qinghai, China	200,000
1932	Gansu, China	70,000
1948	Ashgabat, Turkmenistan	110,000
1970	Peru	66,000
1976	Tangshan, China	255,000
1978	Tabas, Iran	15,000–25,000
1990	Manjil-Rudbar, Iran	35,000–50,000
2001	Gujarat, India	20,000
2003	Bam, Iran	30,000–40,000
2004	Sumatra, Indonesia	220,000
2005	Kashmir, Pakistan	73,000
2008	Sichuan, China	70,000
2010	Haiti	230,000

**Table 2:** Effects of previous earthquakes on adobe buildings [16,17,18].

Earthquake	Casualties	Damaged Masonry Buildings
Van, Turkey, 2011	604	23.33% of buildings heavily damaged and collapsed
Bam, Iran, 2003	30,000–40,000	85% of the infrastructure demolished
Bingöl, Turkey 2003	177	3214 buildings heavily damaged
El Salvador, 2001	1100	150,000
Southern Peru, 2001	81	25,000

**Table 3:** Comparison of physical and mechanical characteristics of Ni-Ti and Fe-based SMAs and stainless steel.

Property	Unit ^a^	Ni-Ti ^b^	Fe-Based	Steel
Density	kg/m^3^	6450–6500	7200–7500	7850
Corrosion resistance	-	Excellent	Good	Fair
Melting point	°C	1260–1310	1320–1350	1510
Poisson’s ratio	-	0.33	0.359	0.265
Young’s modulus	GPa	28–83	160–200	190–193
Specific heat capacity	J/kg °C	450–620	540	420–510
Thermal conductivity	w/m °C	8.6–18	8.4	8.9–16.2
Ultimate tensile strength	MPa	895–1900	680–1200	505
Yield Stress	MPa	70–690	475	215
Recoverable elongation	%	5–10	2.5–13	0.8
Elongation failure	%	5–50	12.4–20	20

^a^ To provide a wide range of information, the values for the given properties are based on the results of different studies as mentioned in the references; ^b^ for Ni-Ti, the range of the given values depends on the crystal phase (martensite or austenite) and other conditions (hardened or fully annealed).

**Table 4:** Energy dissipation of the wall specimens [63].

Energy Dissipation (KJ)							
Number of Cycles	Without SMA	(a)	(b)	(c)	(d)	(e)	(f)
1	0	0	0	0	0	0	0
2	0	0	0	0	0	0	0
3	0	0	0	0	0	0	0
4	0	1	1	0	1	1	1
5	0	2	1	1	4	4	3
6	0	3	1	1	4	4	3
7	11	30	26	26	41	39	36
8	14	28	25	21	39	38	36
9	17	49	35	24	57	54	50
10	62	95	102	122	105	109	114
11	85	114	121	139	132	137	143
12	77	108	111	125	137	137	136
13	137	179	199	206	218	240	271
14	121	158	181	188	210	227	251
Total dissipated energy (KJ)	525	767	803	851	947	988	1044

**Table 5:** The retrofitting methods and their expected outcome of the proposed schemes.

Retrofitting Method	Expected Outcomes	References
SMA-based device between roof and foundation	Higher equivalent modulus of elasticity (*E_eq_*) and energy dissipation might lead to protecting of the URM walls during an earthquake.	[62]
SMA-based device between supportive beam (under the wall) and foundation	Minimize the displacements of URM walls from failure to levels of minor/moderate damage.	[64]
SMA-based strips in the shape of crossings implemented in wall	127.9% increase of the stiffness of the wall and higher energy dissipation capacity.	[63]
SMA-based device between roof and column	Providing a stabilized behaviour during an earthquake.	[58]
SMA-based bolts and connectors	Providing excellent re-centring abilities and moderate energy dissipation capability with an equivalent viscous damping up to 17.5% with 94% deformation recovery.	[74,75]
SMA-based bracing system	Exhibiting greater initial stiffness (resulting in approximately 15% greater initial frequency) with less than half the weight and supplemental re-centring capability, which may lead to sustaining higher inelastic deformations without jeopardizing the structural system’s collapse safety or seismic resistance at the end of the earthquake.	[76]

## Data Availability

The data presented in this article are available on request from the corresponding author.

## References

[1] United Nations Office for Disaster Risk Reduction (UNISDR), Centre for Research on the Epidemiology of Disasters (CRED) (2018). Economic Losses, Poverty and Disasters 1998–2017.

[2] Işik E. (2016). Consistency of the rapid assessment method for reinforced concrete buildings. Earthq. Struct..

[3] Işik E., Kutanis M. (2015). Performance based assessment for existing residential buildings in Lake Van basin and seismicity of the region. Earthq. Struct..

[4] Harirchian E., Kumari V., Jadhav K., Raj Das R., Rasulzade S., Lahmer T. (2020). A Machine Learning Framework for Assessing Seismic Hazard Safety of Reinforced Concrete Buildings. Appl. Sci..

[5] Ghaedi K., Ibrahim Z., Jameel M., Javanmardi A., Khatibi H. (2018). Seismic Response Analysis of Fully Base-Isolated Adjacent Buildings with Segregated Foundations. Adv. Civ. Eng..

[6] Hadzima-Nyarko M., Mišetić V., Morić D. (2017). Seismic vulnerability assessment of an old historical masonry building in Osijek, Croatia, using Damage Index. J. Cult. Herit..

[7] Pavić G., Hadzima-Nyarko M., Plaščak I., Pavić S. (2019). Seismic Vulnerability Assessment of Historical Unreinforced Masonry Buildings in Osijek using Capacity Spectrum Method. Acta Phys. Pol. A.

[8] Javanmardi A., Abadi R., Marsono A.K., Md Tap M., Ibrahim Z., Ahmad A. (2015). Correlation of Stiffness and Natural Frequency of Precast Frame System. Appl. Mech. Mater..

[9] Churilov S., Dumova-Jovanoska E. (2013). In-plane shear behaviour of unreinforced and jacketed brick masonry walls. Soil Dyn. Earthq. Eng..

[10] Ademović N. (2021). Structural assessment & strengthening of the first singe-arch RC bridge in Sarajevo, BIH. Eng. Struct..

[11] Javanmardi A., Ghaedi K., Huang F., Hanif M.U., Tabrizikahou A. (2021). Application of Structural Control Systems for the Cables of Cable-Stayed Bridges: State-of-the-Art and State-of-the-Practice. Arch. Comput. Methods Eng..

[12] Saatcioglu M. (2013). Structural Damage Caused by Earthquakes.

[13] Işık E., Harirchian E., Bilgin H., Jadhav K. (2021). The effect of material strength and discontinuity in RC structures according to different site-specific design spectra. Res. Eng. Struct. Mater..

[14] Matthys H., Noland L. A review of conventional seismic retrofitting techniques for URM. Proceedings of the International Seminar on Evaluation, Strengthening and Retrofitting Masonry Buildings.

[15] Papanikolaou A., Taucer F. (2004). Review of Non-Engineered Houses in Latin America with Reference to Building Practices and Self-Construction Projects.

[16] Blondet M., Garcia G. (2003). Adobe Construction.

[17] Oyguc R., Oyguc E. (2017). 2011 Van Earthquakes: Lessons from Damaged Masonry Structures. J. Perform. Constr. Facil..

[18] Dougangün A., Ural A., Livaouglu R. Seismic performance of masonry buildings during recent earthquakes in turkey. Proceedings of the 14th World Conference on Earthquake Engineering.

[19] Bazazzadeh H., Nadolny A., Attarian K., Safar ali Najar B., Sara Hashemi Safaei S. (2020). Promoting Sustainable Development of Cultural Assets by Improving Users’ Perception through Space Configuration; Case Study: The Industrial Heritage Site. Sustainability.

[20] Mahdavinejad M., Didehban M., Bazazzadeh H. (2016). Contemporary architectural heritage and industrial identity in historic districts, case study: Dezful. J. Stud. Iran. Islam. City.

[21] Javanmardi A., Ibrahim Z., Ghaedi K., Khatibi H. (2017). Numerical analysis of vertical pipe damper. IABSE Symposium, Vancouver 2017: Engineering the Future.

[22] Auroville Introduction to a Millennia Old Tradition. http://www.earth-auroville.com/world_techniques_introduction_en.php.

[23] Kouris L.A.S., Kappos A.J. (2015). Numerical Investigation and Empirical Seismic Vulnerability Assessment of Timber-Framed Masonry Buildings. Handbook of Research on Seismic Assessment and Rehabilitation of Historic Structures.

[24] Hadzima-Nyarko M., Ademovic N., Pavic G., Kalman Šipoš T. (2018). Strengthening techniques for masonry structures of cultural heritage according to recent Croatian provisions. Earthq. Struct..

[25] Elgawady M.A., Lestuzzi P. A review of conventional seismic retrofitting techniques for URM. Proceedings of the 13th International Brick and Block Masonry Conference.

[26] Moghadam A.S., Eskandari A. (2004). Post-earthquake quick inspection of damaged buildings in Bam earthquake of 26 December 2003. J. Seismol. Earthq. Eng..

[27] Biglari M., Formisano A. (2020). Damage Probability Matrices and Empirical Fragility Curves From Damage Data on Masonry Buildings After Sarpol-e-zahab and Bam Earthquakes of Iran. Front. Built Environ..

[28] Maheri M.R. (2005). Performance of Building Roofs in the 2003 Bam, Iran, Earthquake. Earthq. Spectra.

[29] Ghafouri A.M. (2004). Editorial summary: Bam earthquake of 05: 26: 26 Of 26 December 2003, MS6. 5. J. Seismol. Earthq. Eng..

[30] Jasieńko J., Raszczuk K., Kleszcz K., Fra̧ckiewicz P. (2021). Numerical analysis of historical masonry domes: A study of St. Peter’s Basilica dome. Structures.

[31] Ramazi H., Jigheh H.S. (2006). The Bam (Iran) Earthquake of December 26, 2003: From an engineering and seismological point of view. J. Asian Earth Sci..

[32] Ahmadizadeh M., Shakib H. (2004). On the 26 December 2003, southeastern Iran earthquake in Bam region. Eng. Struct..

[33] Hosseini H.B. (2004). Performance of batten columns in steel buildings during the Bam earthquake of 26 December 2003. J. Seismol. Earthq. Eng..

[34] Hosseini Hashemi B., Hassanzadeh M. (2008). Study of a semi-rigid steel braced building damaged in the Bam earthquake. J. Constr. Steel Res..

[35] Langenbach R. (2005). Performance of the Earthen Arg-e-Bam (Bam Citadel) during the 2003 Bam, Iran, Earthquake. Earthq. Spectra.

[36] Bazazzadeh H. Truth of sincerity and authenticity or lie of reconstruction; whom do the visitors of cultural heritage trust?. Proceedings of the International Conference of Defining the Architectural Space.

[37] Bazazzadeh H., Mahdavinejad M., Ghomeshi M., Safaei S.S.H. Requirements for comprehensive management of industrial heritage sites and landscapes. Proceedings of the International Conference on Conservation of 20th Century Heritage from Architecture to Landscape.

[38] Mahdi T. (2004). Performance of traditional arches, vaults and domes in the 2003 Bam Earthquake. Asian J. Civ. Eng..

[39] Ölander A. (1932). An electrochemical investigation of solid cadmium-gold alloys. J. Am. Chem. Soc..

[40] Vernon L.B., Vernon H.M. (1941). Process of Manufacturing Articles of Thermoplastic Synthetic Resins. U.S. Patent.

[41] Buehler W., Gilfrich J., Wiley R. (1963). Effect of low-temperature phase changes on the mechanical properties of alloys near composition TiNi. J. Appl. Phys..

[42] Khachaturyan A.G. (1983). Theory of Structural Transformations in Solids.

[43] Olson G., Owen W. (1992). Martensite. A tribute to Morris Cohen.

[44] Otsuka K., Wayman C.M. (1999). Shape Memory Materials.

[45] Bhattacharya K. (2003). Microstructure of Martensite. Why it Forms and How it Gives Rise to the Shape-Memory Effect.

[46] Lagoudas D.C. (2008). Shape Memory Alloys. Modeling and Engineering Applications.

[47] Wilde K., Gardoni P., Fujino Y. (2000). Base isolation system with shape memory alloy device for elevated highway bridges. Eng. Struct..

[48] Janke L., Czaderski C., Motavalli M., Ruth J. (2005). Applications of shape memory alloys in civil engineering structures—overview, limits and new ideas. Mater Struct.

[49] Müller I., Xu H. (1991). On the pseudoelastic hysteresis. Acta Metall. Mater..

[50] Raniecki B., Lexcellent C., Tanaka K. (1992). Thermodynamic models of pseudoelastic behaviour of shape memory alloys. Arch. Mech..

[51] Auricchio F., Taylor R., Lubliner J. (1997). Shape-memory alloys: Macromodelling and numerical simulations of the superelastic bahavior. Comput. Methods Appl. Mech. Engrg..

[52] Kuczma M., Mielke A., Stein E. (1999). Modelling of hysteresis in two phase systems. Arch. Mech..

[53] Kuczma M., Mielke A. (2000). Influence of hardening and inhomogeneity on internal loops in pseudoelasticity. ZAMM.

[54] Desroches R., Smith B. (2004). Shape memory alloys in seismic resistant design and retrofit: A critical review of their potential and limitations. J. Earthq. Eng..

[55] Cladera A., Montoya-Coronado L.A., Ruiz-Pinilla J.G., Ribas C. (2020). Shear strengthening of slender reinforced concrete T-shaped beams using iron-based shape memory alloy strips. Eng. Struct..

[56] Choi E., hyun Nam T., Cho S.C., Chung Y.S., Park T. (2008). The behavior of concrete cylinders confined by shape memory alloy wires. Smart Mater. Struct..

[57] Burgoyne C. Fibre reinforced polymers–strengths, weaknesses, opportunities and threats. Proceedings of the 9th International Symposium on Fiber Reinforced Polymer Reinforcement for Concrete Structures (FRPRCS-9).

[58] Cardone D., Angiuli R., Gesualdi G. (2019). Application of Shape Memory Alloys in Historical Constructions. Int. J. Archit. Herit..

[59] Chuang S.W., Zhuge Y. (2005). Seismic Retrofitting of Unreinforced Masonry Buildings—A Literature Review. Aust. J. Struct. Eng..

[60] Tabrizikahou A., Nowotarski P. (2021). Mitigating the Energy Consumption and the Carbon Emission in the Building Structures by Optimization of the Construction Processes. Energies.

[61] Shrestha K.C., Araki Y., Nagae T., Omori T., Sutou Y., Kainuma R., Ishida K. (2011). Applicability of Cu-Al-Mn shape memory alloy bars to retrofitting of historical masonry constructions. Earthquakes Struct..

[62] Casciati S., Hamdaoui K. (2008). Experimental and numerical studies toward the implementation of shape memory alloy ties in masonry structures. Smart Struct. Syst..

[63] Rezapour M., Ghassemieh M., Motavalli M., Shahverdi M. (2021). Numerical Modeling of Unreinforced Masonry Walls Strengthened with Fe-Based Shape Memory Alloy Strips. Materials.

[64] Habieb A.B., Valente M., Milani G. (2019). Hybrid seismic base isolation of a historical masonry church using unbonded fiber reinforced elastomeric isolators and shape memory alloy wires. Eng. Struct..

[65] Castellano M.G., Indirli M., Martelli A. (2001). Progress of application, research and development, and design guidelines for shape memory alloy devices for cultural heritage structures in Italy. Smart Structures and Materials 2001: Smart Systems for Bridges, Structures and Highways.

[66] Bruneau M. (1994). State-of-the-Art Report on Seismic Performance of Unreinforced Masonry Buildings. J. Struct. Eng..

[67] Kouris E.G.S., Kouris L.A.S., Konstantinidis A.A., Kourkoulis S.K., Karayannis C.G., Aifantis E.C. (2021). Stochastic Dynamic Analysis of Cultural Heritage Towers up to Collapse. Buildings.

[68] Shabdin M., Attari N.K.A., Zargaran M. (2020). Shaking table study on the seismic performance of an Iranian traditional Un-Reinforced Masonry (URM) building. Structures.

[69] Building and Housing Research Center (2005). Iranian Code of Practice for Seismic Resistance Design of Buildings: Standard No. 2800.

[70] Office of National Building Regulations (2013). Design and Construction of Buildings with Masonry Materials.

[71] Research Center of the Ministry of Roads, Urban Development and Housing (2014). Iranian building standards and regulations. Earthquake Design Regulations—Standard 2800.

[72] Alam M.S., Youssef M.A., Nehdi M. (2007). Utilizing shape memory alloys to enhance the performance and safety of civil infrastructure: A review. Can. J. Civ. Eng..

[73] Song G., Ma N., Li H.N. (2006). Applications of shape memory alloys in civil structures. Eng. Struct..

[74] Fang C., Yam M.C., Lam A.C., Xie L. (2014). Cyclic performance of extended end-plate connections equipped with shape memory alloy bolts. J. Constr. Steel Res..

[75] Ma H., Wilkinson T., Cho C. (2007). Feasibility study on a self-centering beam-to-column connection by using the superelastic behavior of SMAs. Smart Mater. Struct..

[76] Cardone D., Dolce M., Ponzo F.C., Coelho E. (2004). Experimental behaviour of r/c frames retrofitted with dissipating and re-centring braces. J. Earthq. Eng..

[77] Ureche-Trifu C. (2013). Minimal Intervention and Decision Making in Conserving the Built Heritage. Ph.D. Thesis.

[78] Van Roy N., Verstrynge E., Van Balen K. (2015). Quality management of interventions on historic buildings. Struct. Stud. Repairs Maint. Herit. Archit..

[79] Bellomo S.D.M. (2003). The concept of reversibility in the structural restoration of archaeological sites. Adv. Archit. Ser..

[80] Bertolin C., Loli A. (2018). Sustainable interventions in historic buildings: A developing decision making tool. J. Cult. Herit..

